# A fully automated model to form “dry surface biofilms” under optimal dehydration conditions. application to Enterobacteriaceae in healthcare settings

**DOI:** 10.1016/j.bioflm.2025.100312

**Published:** 2025-08-21

**Authors:** Nicolas Jean-Marie, Talyssa Lebielle, Myriam Louisin, Claude Olive, Karine Marion-Sanchez

**Affiliations:** a CHU Martinique, Hygiene and Environment Laboratory, Fort-de-France, Martinique; b University of Antilles, Pointe-à-Pitre, Guadeloupe; c UMR 1058 PCCEI, University of Montpellier, INSERM, University of Antilles, Montpellier, France

**Keywords:** Dry surface biofilm, dried inoculum, Model, Enterobacteriaceae, Methicillin- resistant *Staphylococcus aureus*, XDR *Klebsiella pneumoniae*, Healthcare setting

## Abstract

Over ten years ago, bacteria attached to surfaces and surrounded by extracellular polymeric substances were observed on dry surfaces in intensive care units. These structures were named "dry surface biofilms" (DSBs). Most in vitro models used to study “DSBs” alternate long hydration phases with short periods of desiccation, producing "semi-dehydrated DSBs" that differ from the conditions in healthcare settings.

Our aim was to create a model that could produce "DSBs" under optimal dehydration conditions and apply it to Enterobacteriaceae. These bacteria are commonly responsible for healthcare-associated infections in our hospital, yet they have received little attention in the context of "DSBs." We developed a fully automated model that mimics the splashing of respiratory secretions by repeatedly nebulizing an inoculum of contaminated artificial saliva. Hydration phases lasted 2 s every 6 h. We investigated the microscopic aspect, mean surface coverage, bacterial culturability and membrane integrity.

After validating the model with methicillin-resistant *Staphylococcus aureus* (MRSA), we tested wild-type *Enterobacter cloacae*, wild-type *Klebsiella pneumoniae* and extensively drug-resistant (XDR) *Klebsiella pneumoniae*. The latter formed compact dried inocula with the highest surface coverage (29.7 %), containing curled-up bacteria alongside a low number of culturable cells (3 log_10_). Conversely, dried *S. aureus* inocula covered a lower surface (10.9 %) but contained more culturable cells (6 log_10_), which persisted for more than two months. After several weeks of storage, even the samples containing no more culturable bacteria showed bacteria with intact membranes. Subsequent studies must further assess in depth the composition of these deposits and the viability of the bacteria they contain.

## Highlights

• Spraying bacteria with a nebulizer mimics the projection of respiratory droplets.
• Holes near the coupons, laminar flow, 2-s sprays ensure optimal dehydration.
• Klebsiella forms compact dried inocula with bacteria embedded in a “translucent resin”.
• The higher the coverage, the less culturable the bacteria are.

## 1 Introduction

Since Costerton revealed the link between bacterial biofilms and persistent infections [1], biofilms' involvement in healthcare-associated infections (HAIs) has been thoroughly investigated in the context of invasive, prosthetic, or implantable medical devices; chronic or surgical wounds; urinary and biliary tracts; dialysis machines; water treatment systems; drinking water pipelines; sink drains; sanitary hot water pipelines hosting Legionella [[2], [3], [4], [5], [6], [7], [8]]. Biofilms that form on these surfaces, whether artificial or living, meet the three commonly recognized fundamental criteria for bacterial biofilm growth: bacteria, moisture and interface [3,9]. In the medical field, the ubiquity of biofilms on any non-sterile wetted surface was widely accepted until, in 2012, Vickery and colleagues observed fomites collected from dry surfaces in an intensive care unit. Scanning electron microscopy revealed structures resembling bacterial biofilms [10]. Three years later, the term "dry surface biofilm" (DSB) first appeared in the scientific literature and became the generic term for similar structures found on non-regularly hydrated surfaces [11]. Consequently, the notion of wetted surfaces became relative. Well-known biofilms were called "traditional hydrated biofilms" (THBs) in contrast to this new paradigm [12]. Currently, authors suggest that “DSBs” form on dry hospital surfaces following contamination by biological fluids from patient secretions or the hands of healthcare workers. “DSBs” may be regularly and briefly hydrated by new secretions, contacts with sweaty hands, or detergents and/or disinfectants used for cleaning surfaces. Some in vitro studies describe "DSBs" as being more tolerant to bactericidal products, allowing them to persist on dry surfaces for extended periods despite regular cleaning [11,[13], [14], [15]]. "DSBs" may contain live bacteria, but what makes them particularly insidious is the detection of viable bacteria in culture-negative samples [16]. In healthcare settings, particularly within the immediate patient environment, "DSBs" appear to serve as reservoirs for sessile bacteria, including multidrug-resistant organisms [11,14,16,17]. Some studies have shown that bacteria can be transferred from "DSBs" to the hands or gloves of healthcare workers, which may cause HAIs through cross-transmission [[18], [19], [20]].

To study "DSBs" in vitro, scientists have developed various models. Eleven of these models have recently been identified in the literature [16]. Three derived from the CDC bioreactor, which is commonly used to grow THBs under continuous stirred conditions. Seven were sedimentation models on culture plates, glass coverslips or other test bodies, and one used a drip flow reactor [21]. More than half of these models had long hydration phases followed by periods of desiccation, which led to the development of what some authors called "semi-dehydrated biofilms" [15,16,22,23]. However, these conditions are far from those in healthcare settings, where hydration phases are likely to be extremely short [16,24].

Most models were designed using strains of *Staphylococcus aureus* isolated from dry samples collected in ICUs [10,14,20,25,26]. In Europe, *S. aureus* is the main pathogen identified in bacteremia and surgical site infections [27]. However, in some parts of the world, other strains predominate in healthcare-associated infections (HAIs), such as *Klebsiella pneumoniae* (KPN) in the French West Indies. Currently, very few studies *on Klebsiella pneumoniae* "DSBs" are available in the scientific literature [28,29].

We previously developed a spray-based method that mimicked the splashing of contaminated respiratory secretions. This method involved spraying monobacterial suspensions in sterile artificial saliva onto polyethylene surfaces. However, this method had two major limitations. First, each step was performed manually, resulting in high within-run and between-run coefficients of variation. Second, bacterial accumulation was very slow, requiring 12 days to double the initial inoculum [24].

The main objective of this new study was to improve the spray-based method and use it to create a model that mimics real-life conditions and produces workable materials in a repeatable and reproducible way in less than 12 days. A second objective was to apply the model to various Enterobacteriaceae.

## 2 Methods

### 2.1 Experimental design

**Culture medium.** The culture medium used in the model was Sterile Artificial Saliva (SAS) [24] enriched with 35 % of Brain Heart Infusion® (BHI) (Biomerieux, France). It was autoclaved for 15 min at 121 °C and stored at 4 °C.

**Strains.** Four strains were used in this study. The model was developed and validated using methicillin-resistant *Staphylococcus aureus* (MRSA) ATCC 700698 (CIP 106415). The model was then applied to three Enterobacteriaceae of environmental origin.

- Wild-type *Klebsiella pneumoniae* (KPN), collected from the surface of an ultrasound scanner in a neonatal intensive care unit;
- Extensively drug-resistant NDM-producing *Klebsiella pneumoniae* (XDR-KPN), collected from the shower drain in the onco-hematology department;
- Wild-type *Enterobacter cloacae* (ECLO), collected from the sink drain in the recovery room.

All three strains were part of our institutional collection and stored at −70 °C on glass beads. The strains were tested separately. First, each strain was suspended in BHI and incubated overnight at 37 °C. Then, three successive subcultures were performed in Sterile Enriched Artificial Saliva (SEAS) at room temperature (22 °C ± 2) for 72h.

**Inoculum.** The test strain was cultured for 72 h in SEAS. Then, the culture was diluted by half in SEAS to prepare 6 ml of inoculum at the late exponential phase (approximately 10^9^ CFU/ml).

**Design and operation.** The method is based on the repeated nebulization of an inoculum**.** The model (Fig. 1) is a fully automated system that mimics the contamination of a patient's immediate environment by oropharyngeal secretions. Every 6 h, it sprays contaminated aerosols vertically for 2 s onto 2-cm-diameter coupons. The model is placed under a Class II laminar flow hood (MSC Advantage, Thermo Scientific, France) with a flow rate of 20,000 m^3^/h. An air handling unit equipped with a monitoring system maintains room temperature and humidity at 22 °C ± 2 and 36 % ± 2, respectively.

**Fig. 1:**
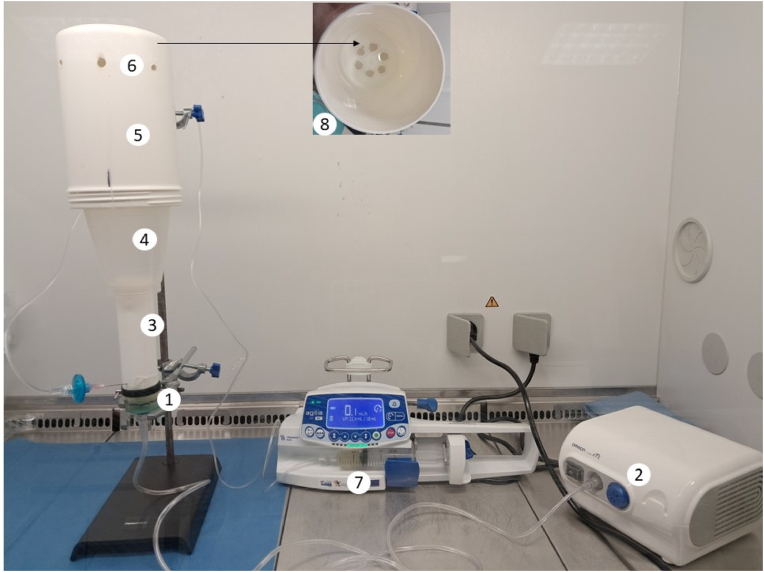
Fully automated spray-based model *1: spray chamber; 2: pneumatic nebulizer; 3: 3.*7 cm *diameter polycarbonate cylinder; 4: polypropylene funnel; 5:* 10 cm *diameter polycarbonate cylinder; 6:* 5 mm *holes; 7: syringe pump delivering uninoculated SEAS; 8: six coupons at the bottom of the* 10 cm *diameter polycarbonate cylinder*.

At the heart of the model is a compressor nebulizer, which is used in medicine for aerosol therapy. The compressor pump generates compressed air, which is then pumped up through the spray chamber containing the liquid to be sprayed. When the air mixes with the liquid and comes into contact with the nozzle, it turns into fine particles and is sprayed. The mass median aerodynamic diameter of the particles generated by the system is 3 μm.

The spray chamber is connected to the nebulizer (OMRON C28P, France) via a PVC tube. A 40 cm high “chimney” is mounted above the chamber to contain and concentrate the flow of particles on the surfaces to be contaminated. A 3.7 cm diameter x 12 cm high polycarbonate cylinder is attached to the top of the spray chamber with sterile adhesive film (IV3000®, Smith & Nephew, England). This cylinder is topped by a 10-cm-diameter sterile polypropylene funnel (Microfil® 250 ml, Millipore, France), which is in turn topped by a 16-cm-high polycarbonate cylinder (10-cm diameter).

Six 2-cm-diameter coupons of transparent, extruded copolyester (Vivak® PET-G sheet, Bayer, Germany) are fixed concentrically to the bottom of the last 16-cm-high polycarbonate cylinder with sterile, removable adhesive gum. Four centimeters from the top, the cylinder is perforated with 5-mm holes spaced 5 cm apart to allow sterile air to enter and facilitate drying.

The spray chamber operates as a chemostat, with a constant supply of nutrients and the regular removal of an equivalent volume every 6 h. At T = 0, it is filled with 6 ml of the suspension to be tested. Every 6 h, 0.6 mL of this suspension is sprayed. Uninoculated SEAS solution is continuously added to the spray chamber at a flow rate of 0.1 ml/h using a syringe pump. Over 24 h, the nebulizer delivers 2.4 mL of bacterial suspension, which is replaced by 2.4 mL of new, uninoculated SEAS. This ensures that the bacterial suspension remains in the late exponential phase throughout the entire experiment. At steady state, μ (h^−1^) = D (h^−1^) = f/v, where μ is the growth rate, D is the dilution rate, f is the flow rate (0,1 ml/h) and v is the total volume of the suspension in the spray chamber (6–6.6 ml from T = 0 to T = 6h), the average growth rate in the spray chamber is therefore 0.016 h^−1^ [30].

The model runs automatically for seven days. Visual checks are performed daily to ensure there are no leaks or turbidity in the uninoculated SEAS, and that there is no variation in the liquid level in the spray chamber.

Components that are not supplied sterile are first cleaned in a 25 % solution of Aniozyme X3 (Anios, France), then disinfected with 70 % alcohol, and finally autoclaved at 121 °C for 15 min. Heat-sensitive coupons and the spray chamber undergo low-temperature sterilization with vaporized hydrogen peroxide (Steris V-PROTM maX, France).

**Temperature and relative humidity monitoring.** A wireless Temperature/Hygrometry data logger (KISTOCK KH-210-AO-RF Kimo-Instruments, France) was used to monitor the temperature and relative humidity inside de model. The device was placed on top of the funnel in the center of the polycarbonate cylinder. The parameters were recorded continuously for 48 h.

### 2.2 Analysis, calculations and statistics

**Percentage of mean surface coverage (% MSC).** The coupons were stained with Crystal Violet according to the method described by Christine et al. [24] and mounted on a glass slide with sterile, removable adhesive gum. They were observed using an optical microscope (DM 1000 LED Leica, France) equipped with an ICC camera (Leica ICC 50 E). Forty images were taken by scanning the coupon field by field from the center in the four cardinal directions (10 images per direction). Each image was analyzed using Scion image software (Scion Corp, USA). In brief, a grayscale copy of the image is generated, and a red/grayscale filter is applied to separate the colored biomass from the background, using a user-defined intensity cutoff. Then, only the surface area covered by the red biomass is measured and expressed in cm^2^. The mean surface coverage (MSC) is calculated for N = 40. In parallel, total surface coverage (TSC) is measured. TSC, a constant value, is the estimated coverage value if the entire surface of the coupon were covered with biomass. The supplementary material 1 provides details and illustrations.

% MSC is calculated as follows: % MSC = (MSC/TSC) X 100.

**Bacteria recovery and culturable cells enumeration.** Coupons coated with dried inocula were rinsed with sterile water to remove free cells. Then, the entire surface was scraped for 3 min with a sterile stainless-steel spatula into 1 mL of phosphate-buffered saline (PBS). Any agglomerated bacteria were disaggregated by passing the resulting suspension three times through the needle (18G, 1.2 mm ID) of a syringe and vortexing it for 1 min. Culturable bacteria were enumerated in the resulting suspension on appropriate culture media after serial dilution in sterile water. Results are given in CFU/cm^2^.

**Viability assessment.** Bacterial viability was assessed using the LIVE/DEAD BacLight® Bacterial Viability Kit (Molecular Probes, Fisher Scientific, France). The entire surface of the coupons was coated with 400 μL of PSB containing 0.3 % of each fluorescent nucleic acid dye (Syto 9 and propidium iodide) for 15 min. Then, the coupons were rinsed with sterile water and observed with an epifluorescence microscope (DM 2500 LED, Leica, France). Viable bacteria with intact membranes fluoresced green, while dead bacteria with damaged membranes fluoresced red.

**Scanning Electron Microscopy.** After fixation with a 2 % formaldehyde solution in a 0.2 M cacodylate buffer, dehydration in alcohol, and metallization with gold/palladium, the samples were observed using scanning electron microscopy (Nova NanoSEM 200 or Quanta 250, FEI, France).

**Bias control.** To avoid selection bias when analyzing coupons, they were selected via online randomization using the dcode.fr website (www.dcode.fr). To avoid interpretation bias, experienced scientists carried out or supervised the image analyses for calculating %MSC.

**Statistics.** The average percentage of MSCs for each strain was compared using a Student's t-test. Significance was defined as p < 0.05. The analysis was performed using OpenEpi software. (www.openepi.com).

## 3 RESULTS

**Fig. 2:**
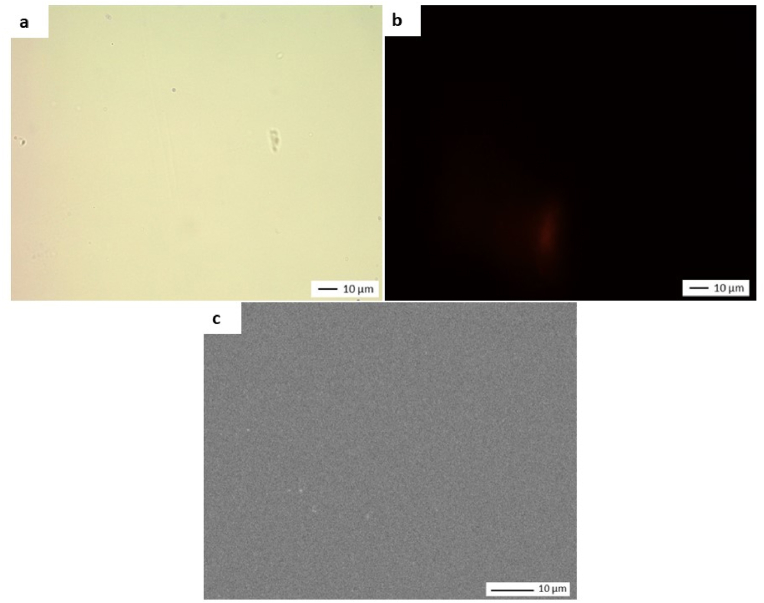
Microscopy images of negative controls *a) Staining with crystal violet and observation with an optical microscope (X 500); b) Live/dead staining and observation with epifluorescence microscope (X 500)*; *c) SEM observation (X 3000).* (For interpretation of the references to color in this figure legend, the reader is referred to the Web version of this article.)

**Negative controls.** Three clean, sterile, and untreated coupons were analyzed as negative controls. As shown in Fig. 2, no confluent or isolated bacteria were observed on the surface of the untreated coupons using either optical microscopy after staining or SEM. Only a few scattered machining residues, not removed by preliminary cleaning, were visible. No fluorescence was observed after Live/Dead staining.

The average percentage of MSCs obtained by analyzing three untreated coupons was 0.04 % (±0.0007), which is four times the method's limit of quantification of 0.01 %.

No culturable cells were isolated from three untreated coupons.

**Validation of drying conditions.** The monitoring of the temperature and relative humidity inside the model is presented in Supplementary Material 2. During the 48-h observation period, the temperature remained stable at 22 °C ± 1, and the relative humidity fluctuated slightly between 63 % and 67 % with each nebulization step. Despite an artifact at the beginning of the monitoring period, the relative humidity remained around 65 %.

**Inoculum stability in the spray chamber.** The number of culturable cells in the spray chamber was counted at time zero (T0) and at the end of the experiment (day 7) in three independent assays. The results are given in Table I.

**Table 1:** Culturable cells (CFU/ml) inside the spray chamber at T0 and the endpoint.

	T0			Day 7 (endpoint)		
Assay n°	1	2	3	1	2	3
CFU/ml	2.0 × 10^9^	1.2 × 10^9^	1.2 × 10^9^	3.5 × 10^9^	1.4 × 10^9^	2.7 × 10^9^
Mean (N = 3) ( ± SD)	**1.4 x 10^9^** ( ± 4.6 × 10^8^)			**2.5 x 10^9^** (±10^9^)		

Despite a slight increase in the number of culturable cells counted at the end of each assay, the variation between the T0 and endpoint values is minimal (0.25 log_10_), indicating stable inoculum throughout the assay. Note that visible agglomerates formed rapidly inside the culture (within less than 24 h) and persisted throughout the assay.

**Fig. 3:**
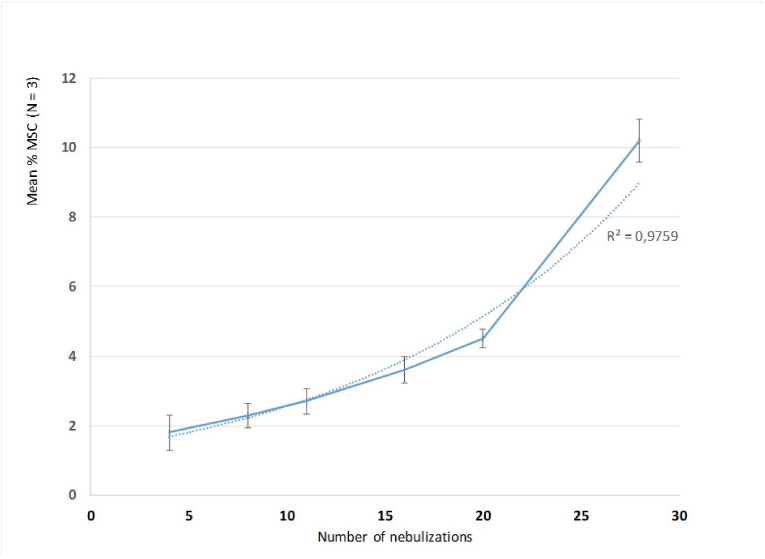
MRSA accumulation kinetic curve. *The curve is drawn as a solid line (means* ± *standard deviation). The dotted line represents the exponential trend*.

**Multi-Resistant *Staphylococcus aureus* (MRSA) accumulation kinetic curve.** Three independent assays were carried out. For each assay, one coupon was collected and analyzed every four nebulizations. Average % MSC (N = 3) was calculated at each sampling time. The MRSA accumulation kinetic curve (percentage of surface coverage as a function of the number of nebulizations), is shown in Fig. 3 (one day equals four nebulizations).

From 4 to 28 nebulizations, bacterial accumulation follows an exponential curve (R^2^ = 0.978). Accumulation speed is slow until day 5 (20 nebulizations) and accelerates from day 5–7.

**Fig. 4:**
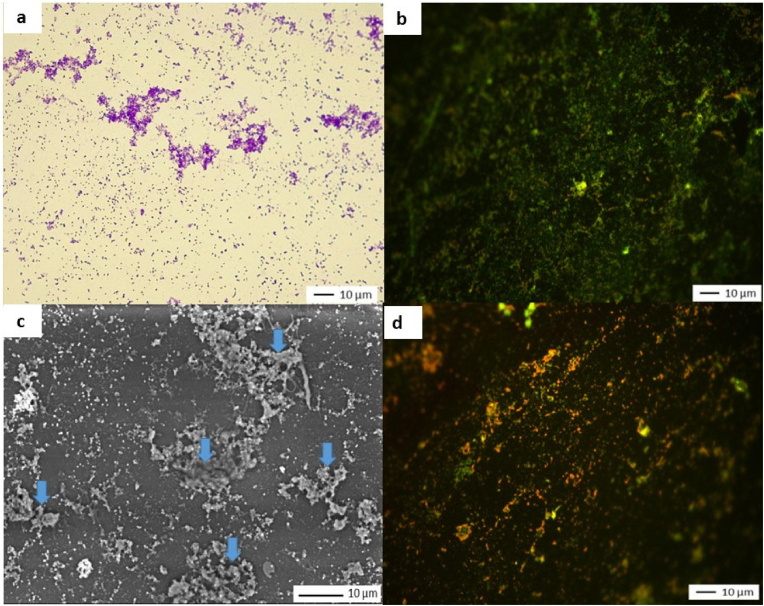
Microscopy images of coupons covered with MRSA *a) Crystal violet staining and light microscop*y *o, J7 (X 500); b) Live/Dead staining and epifluorescence microscopy, J7 (X 500); c) SEM observation, J7 (X 2500), blue arrows indicate extracellular substances; d) Live/Dead staining and epifluorescence microscopy (after* 3 months *of storage) (X 500)*. (For interpretation of the references to color in this figure legend, the reader is referred to the Web version of this article.)

**Microscopic observation of the surface at day 7**. Fig. 4 shows microscopy images of the surface of the coupons after 28 nebulizations (images a, b and c). Images a and c show isolated or confluent adherent cells with heterogeneous distribution across the entire surface. No area is free of bacteria. Image b shows green fluorescence, indicating the presence of bacteria with intact membranes. The SEM image (c) shows isolated and confluent cells, as well as areas covered by extracellular substances (indicated by blue arrows).

**Repeatability (within-run variation).** Table II shows the percentages of MSC obtained on day 7 from each of the six coupons during a single experiment. The average percentage of MSCs was 10.7 % ± 1.3 % (n = 6), yielding a coefficient of variation of 12.1 %.

**Table 2:** Within-run variation.

Coupon n°	C1	C2	C3	C4	C5	C6	Average (N = 6)	SD	CV (%)
% MSC	9.5	13.0	11.2	10.2	10.7	9.6	**10.7**	1.3	**12.1**

**Reproducibility (between-run variation).** Table III shows the percentages of MSC obtained on day 7 from three coupons in three independent experiments carried out by three different operators. The average percentage of MSCs was 9.3 % ± 1.7 % (n = 9), giving a coefficient of variation of 13.7 %.

**Table 3:** Between-run variation.

	Operator 1			Operator 2			Operator 3			Average (N = 9)	SD	CV (%)
Coupon n°	C1	C2	C5	C3	C4	C5	C3	C4	C6			
% MSC	9.1	13.0	10.7	8.6	8	9.6	8	8	8.1	**9.3**	1.7	**13.7**
Average (N = 3) ( ± SD)	11.1 ( ± 1.8)			8.7 ( ± 0.8)			8.0 ( ± 0.06)					

**Culturability and validation of bacterial recovery.** Respectively 8.6 × 10^6^ and 6.7 × 10^6^ CFU/cm^2^ were recovered from two randomly selected coupons with an average of 7.6 × 10^6^ ± 1.3 × 10^6^ CFU/cm^2^. After scraping, only a few small residues were visible on the surface of the coupons, showing that most fixed bacteria had been detached. Light microscopy, SEM, and Live/Dead images are presented in Supplementary Material 3.

Data from the analysis of images of coupons before (% MSC = 9.8 %) and after scraping (% MSC = 0.6 %) (mean, N = 2) indicate that the method removed 93.9 % of the initial coverage.

**MRSA survival.** Two independent assays were conducted to study MRSA survival after storing the coupons at room temperature (22 °C ± 2 °C) with a relative humidity of 55 %–65 %. Each month, two coupons were scraped to count culturable bacteria. The results are shown in Table IV. When no culturable cells were counted, live cells were sought using Live/Dead staining (Fig. 4, Image d).

**Table 4:** Residual culturable cells.

Month	T0 (N = 2)	M1 (N = 2)	M2 (N = 2)	M3 (N = 2)
Mean CFU/cm^2^	7.6 × 10^6^	2.2 × 10^4^	4.1 × 10^3^	<1
Loss in culturability^a^ (log^10^)	–	2.6	0.7	3.6

a Compared with the previous measure.

***Klebsiella pneumoniae* (KPN).** Three independent assays were performed with wild-type KPN. For each of them, the number of culturable cells remained stable in the spray chamber for 7 days but visible agglomerates formed rapidly inside the culture (<24 h) and persisted throughout the assay.

**Fig. 5:**
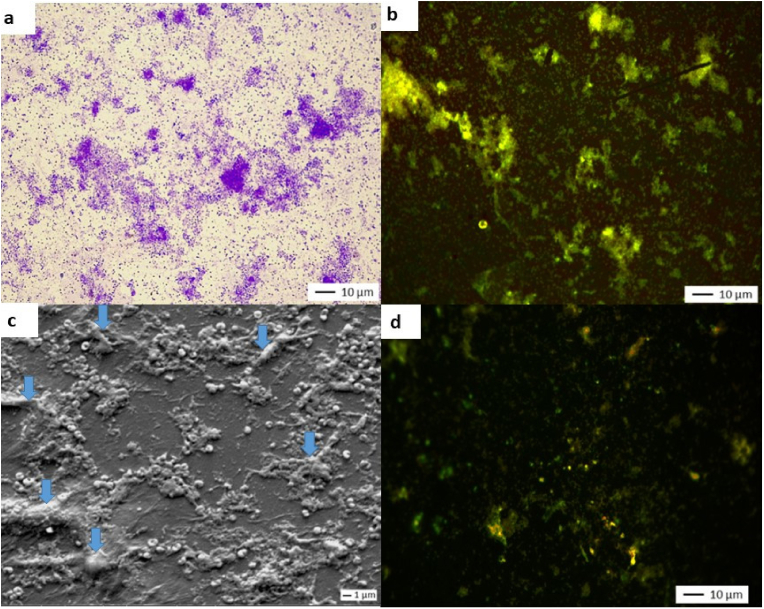
Microscopy images of coupons covered with KPN *a) Crystal violet staining and light microscop*y*, J7 (X 500); b) Live/Dead staining and epifluorescence microscopy, J7 (X 500); c) SEM observation, J7 (X 7000), blue arrows indicate extracellular substances; d) Live/Dead staining and epifluorescence microscopy (after* 1 month *of storage) (X 500)*. (For interpretation of the references to color in this figure legend, the reader is referred to the Web version of this article.)

Fig. 5 shows images obtained while applying the three microscopic analysis protocols.

Images a and c show primarily isolated or confluent adherent cells. The cells are distributed heterogeneously across the entire surface, but no area is free of cells. Light microscopy and SEM observations indicate a change in bacterial morphology. These bacilli bacteria appear in a "cocci-like" form with a curled-up appearance. Some areas are covered by extracellular substances, visible on SEM images (blue arrows). The green fluorescence visible on image b indicates the presence of bacteria with intact membranes.

Quantitative analysis revealed a % MSC of 20.4 ± 5 and a level of culturable bacteria of 3.1 × 10^4^ CFU/cm^2^ (average, n = 3). After one month of storage under ambient conditions (22 °C ± 2 °C; relative humidity 55 %–65 %), no bacteria remained culturable. However, Live/Dead staining at month 1 showed residual green fluorescence, indicating the presence of bacteria with intact membranes (Fig. 5, image d).

Unlike *Staphylococcus aureus*, which can remain culturable for over three months without a nutrient or moisture supply, KPN seems to rapidly enter a non-culturable state.

***XDR Klebsiella pneumoniae* (XDR KPN).** Three independent assays were performed using extensively drug-resistant (XDR) *Klebsiella pneumoniae*. For each of them, the number of culturable cells remained stable in the spray chamber for 7 days but visible large and viscous agglomerates formed rapidly inside the culture (<24 h) and persisted throughout the assay.

**Fig. 6:**
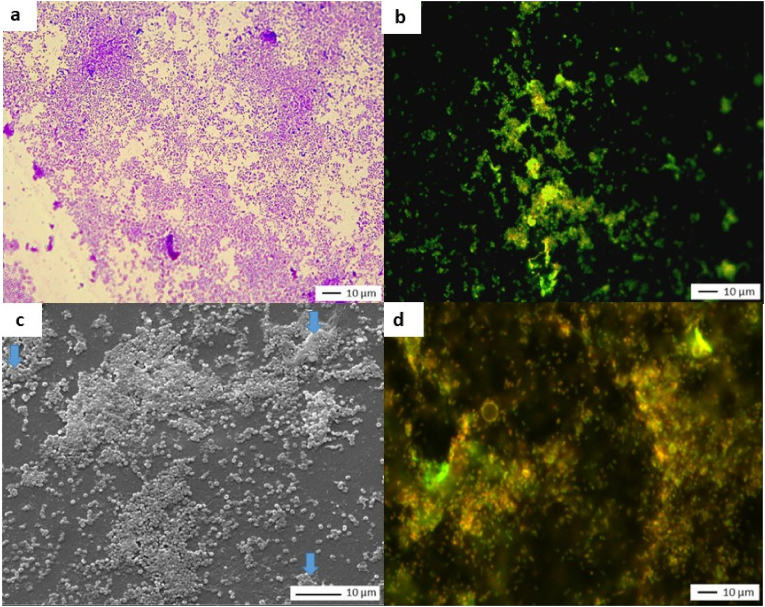
Microscopy images of coupons covered with XDR KPN *a) Crystal violet staining and light microscopy, J7 (X 500); b) Live/Dead staining and epifluorescence microscopy, J7 (X 500); c) SEM observation, J7 (X 3000), blue arrows indicate extracellular substances; d) Live/Dead staining and epifluorescence microscopy (after* 1 month *of storage) (X 500)*. (For interpretation of the references to color in this figure legend, the reader is referred to the Web version of this article.)

Fig. 6 shows images obtained while applying the three microscopic analysis protocols.

**Fig. 7:**
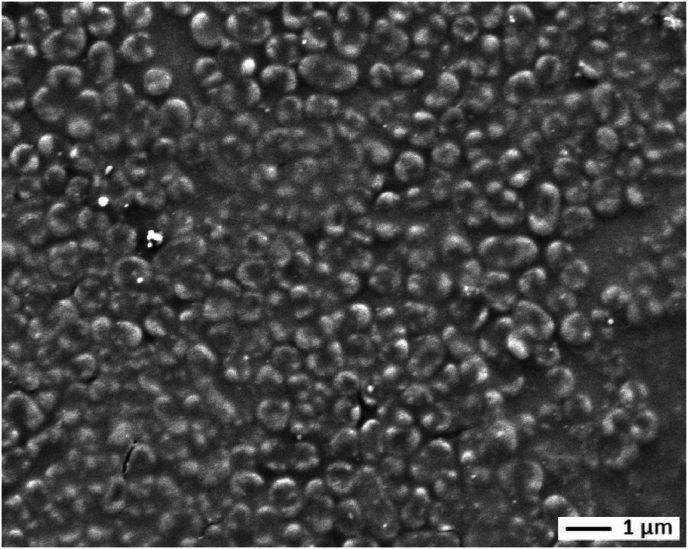
Curled-up appearance of XDR KPN on the surface (SEM, X 9000) *Compact structure made of bacterial cells embedded in a kind of “translucent resin”*.

Although images of KPN and XDR KPN-coated surfaces show similarities, the distribution of XDR KPN is more homogeneous due to increased confluence of adherent cells (images a and c). Very few areas covered with extracellular substances are visible in the SEM image (blue arrows). At higher magnification, the dehydrated cells appear curled up and embedded in a dry, translucent "resin" that gives the structure a compact appearance (Fig. 7). This can also be observed with wild-type KPN DSBs, but it is more pronounced with XDR KPN.

Quantitative analysis revealed a % MSC of 29.7 ± 2.7 and a level of culturable bacteria of 1.1 × 10^3^ CFU/cm^2^ (average, n = 3). After one month of storage under ambient conditions, no bacteria remained culturable, though Live/Dead staining showed residual green fluorescence, indicating the presence of cells with intact membranes (Fig. 6, image d).

**Enterobacter cloacae (ECLO).** Three independent assays were performed using wild-type *Enterobacter cloacae.* For each of them, the number of culturable cells remained stable in the spray chamber for 7 days but visible agglomerates formed rapidly inside the culture (<24 h) and persisted throughout the assay.

**Fig. 8:**
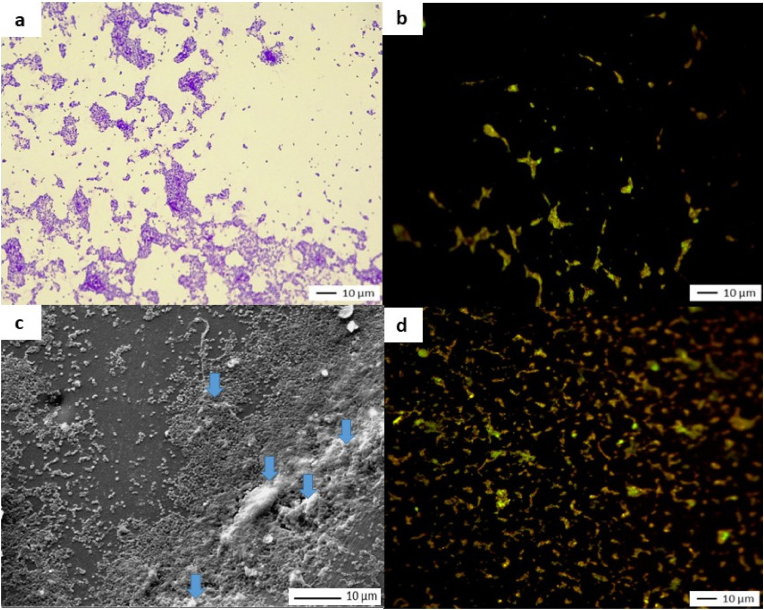
Microscopy images of coupons covered with ECLO *a) Crystal violet staining and light microscopy, J7 (X 500); b) Live/Dead staining and epifluorescence microscopy, J7 (X 500); c) SEM observation, J7 (X 2500, blue arrows indicate extracellular substances); d) Live/Dead staining and epifluorescence microscopy (after* 2 months *of storage) (X 500)*. (For interpretation of the references to color in this figure legend, the reader is referred to the Web version of this article.)

Fig. 8 shows images obtained while applying the three microscopic analysis protocols.

Clusters of confluent adherent cells are primarily visible in image a. Their distribution is heterogeneous across the entire surface, but no area is devoid of bacteria. The curled-up appearance of the bacteria is less pronounced than with KPN. Numerous areas are covered with extracellular substances (image c). Green fluorescence indicates the presence of bacteria with intact membranes (image b).

Quantitative analysis revealed a % of MSC of 15.5 ± 1.7 and a level of culturable bacteria of 2.5 × 10^3^ CFU/cm^2^ (average, n = 3). After one month of storage under ambient conditions, the residual culturable bacteria level was 3 CFU/cm^2^. After two months, no bacteria remained detectable, though Live/Dead staining showed residual green fluorescence indicating the presence of viable bacteria (Fig. 8, image d).

### 3.1 Statistical comparisons of % MSC between the four tested strains

Statistical comparison of the average percentages of MSCs shows a significant difference between each strain (p < 10^−4^), with in descending order: XRD KPN > KPN > ECLO > MRSA.

## 4 Discussion

Under optimal drying conditions, our fully automated model enables the accumulation of dried inocula on surfaces within seven days. The total duration of the hydration phases is only 8 s per day. This model can handle six samples simultaneously and provides repeatable and reproducible results. Due to the "upside-down" position of the coupons, bacterial attachment occurs under the most unfavorable conditions (desiccation, starvation, low temperature, and no sedimentation possible), ensuring that "what sticks, sticks really."

Assembling the model components is quite easy and rapid. The "chimney" guides the droplets from the spray chamber to the coupons over a predefined distance, projecting the finest and most homogeneous droplets possible. This contributes to the reproducibility of the spray on the surfaces of the six coupons. Drying is facilitated by holes drilled close to the coupons and by the airflow from the laminar flow hood.

Since most studies involving clinical dry samples were conducted in intensive care units (ICUs) [16], our model was developed to simulate the projection of contaminated respiratory droplets or aerosols produced by coughing, sneezing, intubation, extubation, or aspiration in ICUs. It can also simulate other fluid projections by changing the medium's composition. Moreover, by changing the material of the coupon, it is possible to test any material for its ability to bind bacteria. The formation of monobacterial or multibacterial deposits is possible if mixed species are inoculated into the spray chamber.

In line with the majority of other studies, the model was developed and validated using *Staphylococcus aureus*. Additionally, numerous authors have emphasized the presence of multidrug-resistant organisms in "DSBs," prompting our decision to work with MRSA [16]. We selected the ATCC 700698 strain, which was isolated from human lungs. This strain has already demonstrated its ability to grow in respiratory secretions and form "THBs” [31] For our study, we targeted Enterobacteriaceae to reflect our tropical specificity. We tested the two strains most commonly responsible for healthcare-associated infections in our hospital, *Klebsiella pneumoniae* and *Enterobacter cloacae*. We selected environmental strains that had previously contaminated surfaces. We added a third strain, XDR *Klebsiella pneumoniae*, to observe the behavior of highly resistant strains on dry surfaces.

We created this model to better reproduce “real life” conditions, but what is “real life”?

The concept of "dry surface biofilms" (DSBs) is still not clearly defined [32]. The term was first introduced by Hu et al., who observed bacterial structures on dry clinical samples collected in ICUs. These samples showed bacteria attached to surfaces and surrounded by EPS; only 52 % contained culturable bacteria but even after 12 months of storage, all of them still showed bacteria with intact membranes, revealed by Live/Dead staining. At the time, the composition of EPS in “DSBs” was unknown [11].

Later, Almatroudi et al. identified proteins, glycoconjugates, and DNA in the EPS using specific stains and confocal laser scanning microscopy [33]. To our knowledge, no further research has been conducted on the EPS composition in clinical dry samples since then.

Three years later, Ledwoch et al. provided a more in-depth analysis, showing that” DSB” samples are highly variable, with small bacterial aggregates irregularly distributed and inconsistent EPS content [14].

Currently, there is no solid scientific evidence that “DSBs” observed in healthcare settings fit the definition of a biofilm. While Hu's description resembles Costerton's classic definition of biofilms [1], there is no proof that “DSB” bacteria grow on dry surfaces to form organized communities, produce EPS by themselves, develop reversible tolerance to biocides, remain persistent on surfaces. This is likely why Sauer et al. did not mention them in their recent review of all known types of biofilms. [34]. It is possible that “DSBs” are merely the accumulation of regular deposits of contaminated fluids that gradually dehydrate and leave bacteria embedded in “dry EPS-like substances” and no more.

At this stage of our work, we can conclude that our model produces an accumulation of dried inocula, composed of isolated and confluent adherent bacteria, some of which are covered by extracellular substances. This resemble “DSBs” described by Hu [11], meeting key characteristics such as attachment to surfaces, culturability and presence of bacteria with intact membranes after storage. This may also align with Ledwoch's criteria (random clusters with uneven EPS distribution) [14]. However, we do not yet know the exact nature or origin of the extracellular substances observed on bacterial deposits. These substances may be produced by bacteria once they are attached to the coupons or may be present in the culture medium and sprayed with the bacteria.

Dried inocula are currently used to study bacterial survival in dry environments and to evaluate the effectiveness of bactericidal treatments on inert surfaces. Most studies use a large drop of bacteria washed from their culture medium and resuspended in sterile water or 0.9 % NaCl. The bacteria are deposited once on a surface and left to dry [35,36]. In our study, bacterial cells were maintained in the late exponential phase of their culture media, which was sprayed with them as tiny droplets 28 times over seven days. In the spray chamber, we observed agglomerates that formed rapidly in all cultures and persisted throughout the assay. These agglomerates were regularly resuspended by the shaking generated through repeated nebulizations. They could be partially disrupted by the shear forces generated during these stages. They may form in the suspension or result from the detachment of secondary, wall-adhered biofilms that form on the surface of the spray chamber during the assay [30].

This study focuses solely on the surface of the coupons but a thorough examination of what is happening inside the spray chamber during the seven days of operation is necessary. Given that each of the species we have studied has been shown to be able to form aggregates [[37], [38], [39]] and knowing that aggregates can form in human saliva [34], we speculate that our sprayed inoculum contains "suspended biofilm-like aggregates" along with planktonic bacteria. Our new goal is to identify the agglomerates present in our bacterial suspensions and on our coupons. If they meet the structural, growth, and phenotypic criteria to be defined as "aggregates" [34], then we will get closer to the concept of “dry surface biofilms.” Similar processes could occur in real life: bacterial aggregates formed in human fluids would be projected or deposited on surfaces, where they would dry.

The appearance of dried KPN inocula, particularly XDR KPN, is noteworthy. The curled-up form has already been described when KPN is in a viable but nonculturable state [40]. It can also be observed in SEM pictures of desiccated "DSBs" in Centelegue's study [29]. However, the compact appearance of the bacteria trapped in a kind of dry, transparent “resin,” has not yet been reported. During our experiments, we observed large, slimy agglomerates in XDR KPN suspensions. These agglomerates may be sprayed with the bacterial inoculum simultaneously and may contribute to the compact appearance of the deposits after drying.

All the dried inocula that we produced contain culturable bacteria at day 7. The levels of culturable bacteria in dried MRSA inocula (6 log10) are close to those in “semi-dehydrated biofilms” produced in vitro by other authors (6.9 log10) [15,22,33,41]. They are lower in dried KPN inocula (3–4 log10 versus 5 - 8 log10 [28,29]), with XDR KPN being the least culturable strain under our experimental conditions. These results suggest that hydration level has a lesser influence on the culturability of MRSA than Enterobacteriaceae.

On the other hand, statistical comparisons of the percentages of MSC revealed that Enterobacteriaceae, particularly XDR KPN, produced more substantial deposits than MRSA in terms of surface coverage. Interestingly, the more a strain covers the surface, the less culturable it is. There appears to be an inverse proportional relationship between dry inocula accumulation and bacterial survival in a culturable form. Further comparisons of wild-type, resistant and highly resistant strains are needed to better understand these observations.

We selected % MSC as one of the primary metrics for our study because it is suitable for analyzing dried inoculum contamination and offers a comprehensive view of bacterial distribution across the entire sample surface. It is both qualitative and quantitative, fast and simple to measure, cost-effective, and highly sensitive. The detection limit corresponds to a single adherent cell.

Since dry samples taken from intensive care units do not systematically contain culturable bacteria [16], the enumeration of culturable bacteria should be supplemented by other metrics to achieve a complete analysis of contaminated dry surfaces. Most of these samples contain bacteria with intact membranes that are presented as viable. The same observation has been made in vitro, regardless of the model used [16], as well as in KPN "DSBs" by Centeleghe et al. [29] and now by ourselves in our dried inocula. Furthermore, the term "viable but nonculturable" (VBNC) is emerging in articles related to "DSBs” [16].

According to Pan et al., VBNCs lose their ability to grow in current culture media. However, their gene expression capacities and metabolic activities remain, albeit diminished. Their cytoplasmic membrane remains intact, though they may exhibit abnormal morphology. They could "resuscitate" when environmental conditions become favorable, regaining metabolic activity and culturability [42]. All of this must be demonstrated to confirm the presence of VBNC bacteria on dry surfaces. This is the research focus that we will explore in greater depth.

Since routine surface monitoring involves enumerating culturable bacteria, "DSBs" appear to be a kind of "chameleon lifestyle," enabling bacteria to evade human surveillance. In line with Centeleghe's conclusions, methods other than fluorescent staining must be developed to quantify non-culturable cells [29], as they may be at the heart of the problem associated with "DSBs" in healthcare facilities.

The model we developed presents a new concept for how "DBSs" could possibly form in healthcare facilities. Because this field of research is still in its infancy, many questions remain unanswered. The first of these is the definition of the structures themselves. The next steps will be to study their composition, growth, viability, evolution over time, tolerance to biocides, transferability to medical devices, and possibilities for removal. Once defined, the choice of metrics best suited to their nature must also be considered. Thorough observations and analyses of real-life samples, as well as numerous in vitro studies, are needed to explore “DSBs”, and it will be many years before we solve their mystery.

## CRediT authorship contribution statement

**Nicolas Jean-Marie:** Writing – original draft, Methodology, Formal analysis. **Talyssa Lebielle:** Writing – original draft, Formal analysis. **Myriam Louisin:** Resources, Formal analysis. **Claude Olive:** Visualization, Validation, Resources. **Karine Marion-Sanchez:** Writing – review & editing, Supervision, Funding acquisition, Data curation, Conceptualization.

## Declaration of competing interest

The authors declare that they have no known competing financial interests or personal relationships that could have appeared to influence the work reported in this paper.

## Figures and Tables

**figs1:**
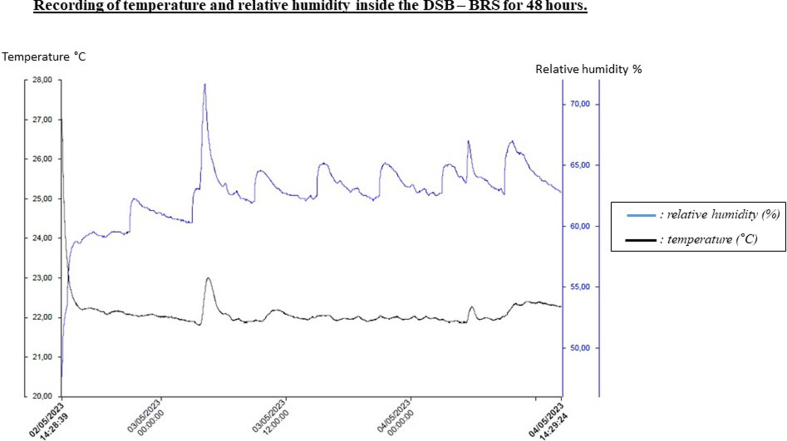


## Data Availability

Data will be made available on request.
