# Supplementary material for: A fully automated model to form “dry surface biofilms” under optimal dehydration conditions. application to Enterobacteriaceae in healthcare settings
Source: Biofilm. 2025 Aug 21;10:100312. doi: 10.1016/j.bioflm.2025.100312 (PMC12745990; doi:10.1016/j.bioflm.2025.100312)
Supplement: Multimedia component 1 [file mmc1.docx]

**Coverage measurement using Scion Images software**

Image analysis is performed with “slice density” option and “analyze” tool.

**Step 1:** original image (.tif) obtained with light microscopy after staining with crystal violet

**
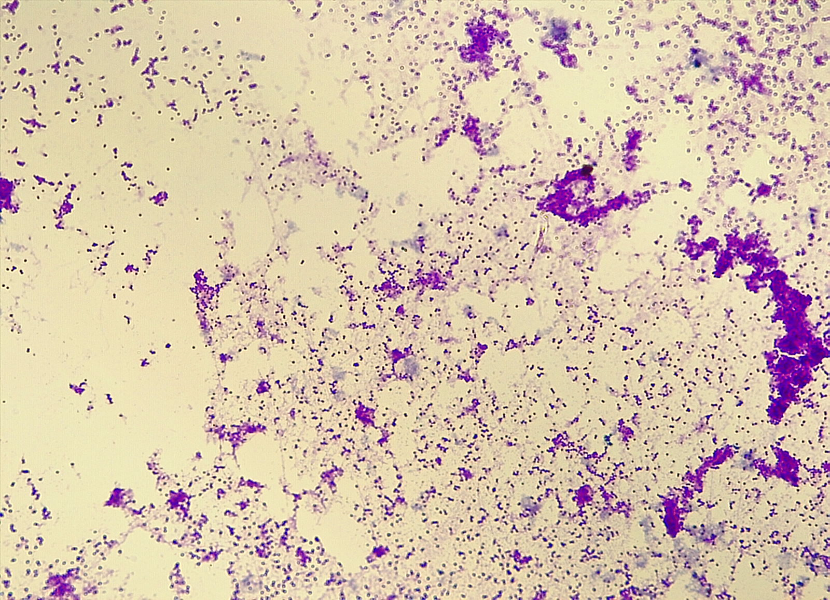
**

**Step 2:** Scion images opens the original image together with a grayscale copy


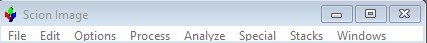


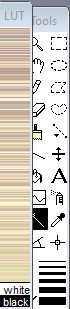

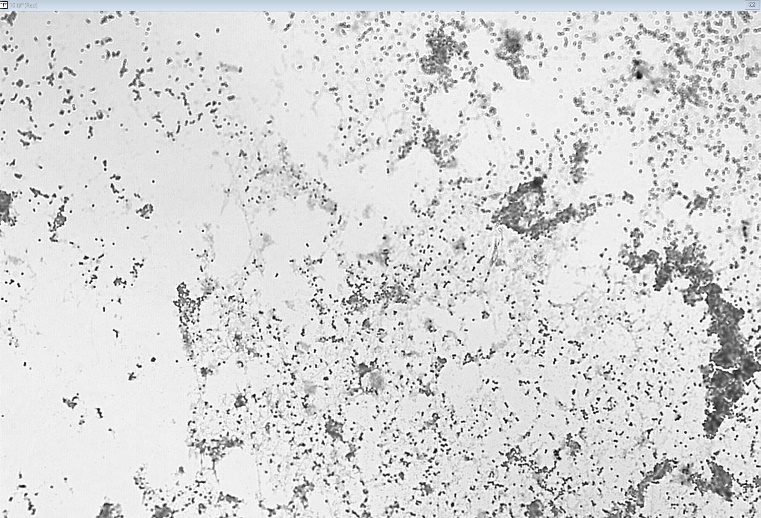


**Step 3:** The “density slice” option uses a red/gray scale to separate the colored biomass (red) from the surface background (grey). Intensity cutoff is defined by the user.


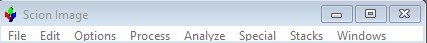


**
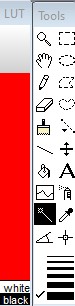

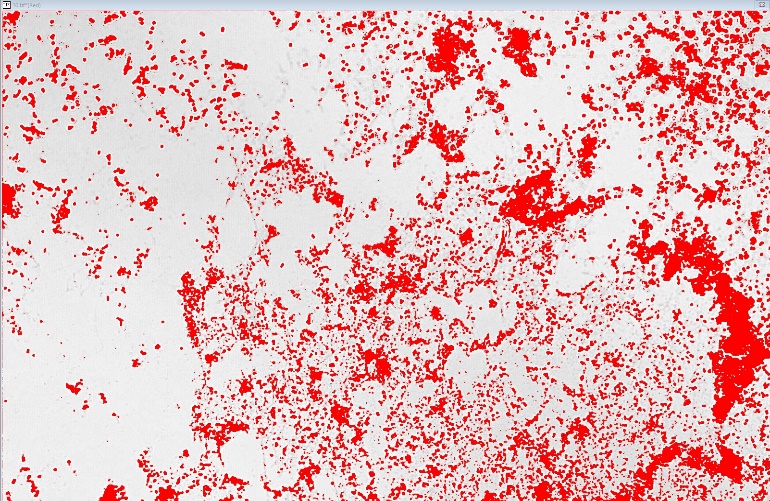
**

**Step 4:** The “analyze” tool measures exclusively the surface covered by the red colored biomass, here **260 cm²**


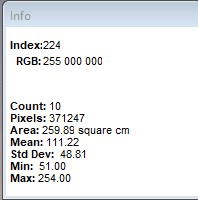


**Step 5:** Total surface coverage is measured and equals **1343 cm²**


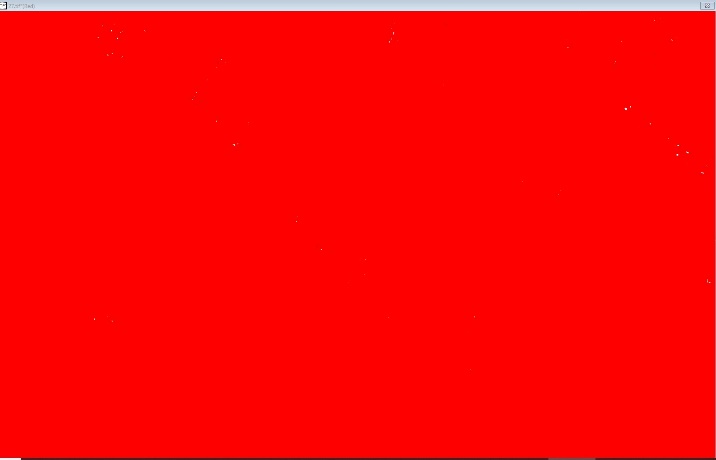

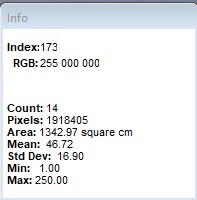


**Step 6**: Percentage of surface coverage = (**260/1343) x 100 = 19%**
