# Supplementary material for: A fully automated model to form “dry surface biofilms” under optimal dehydration conditions. application to Enterobacteriaceae in healthcare settings
Source: Biofilm. 2025 Aug 21;10:100312. doi: 10.1016/j.bioflm.2025.100312 (PMC12745990; doi:10.1016/j.bioflm.2025.100312)
Supplement: Multimedia component 2 [file mmc2.docx]

**Effectiveness of the method used to recover dried MRSA inocula**

**from the surface of the coupons**

1. **Microscopy images**

Microscopy images show very few residues after scraping the surface of coupons covered with dried MRSA inocula.


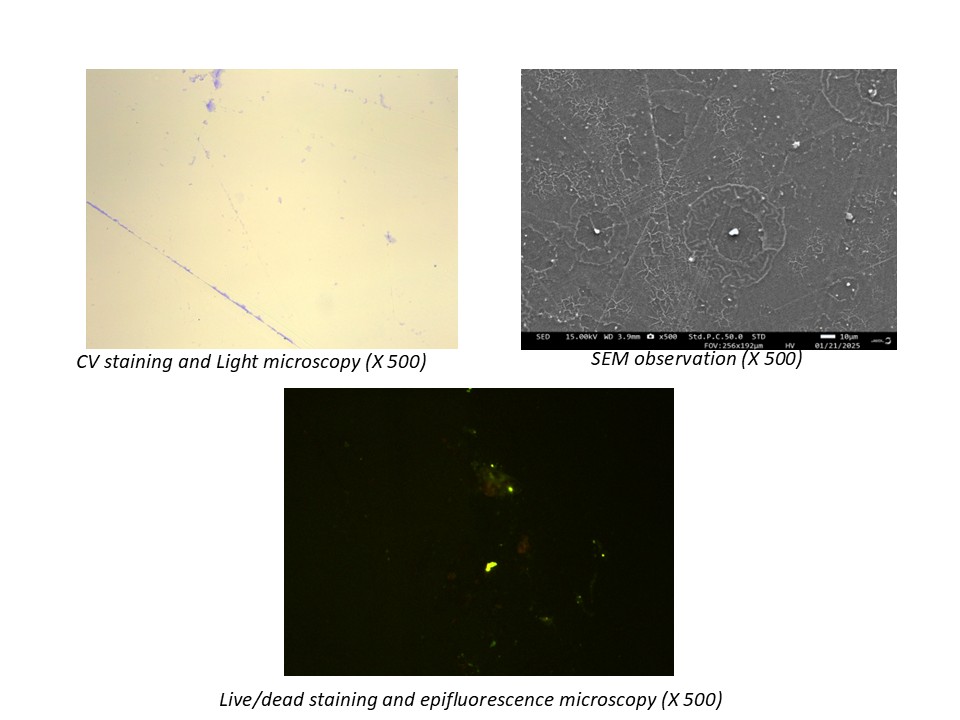


1. **Quantitative data**

Enumeration of culturable cells in the suspension shows that the disaggregation techniques do not kill the bacteria. Conversely, disaggregation slightly increases the number of culturable cells.

**
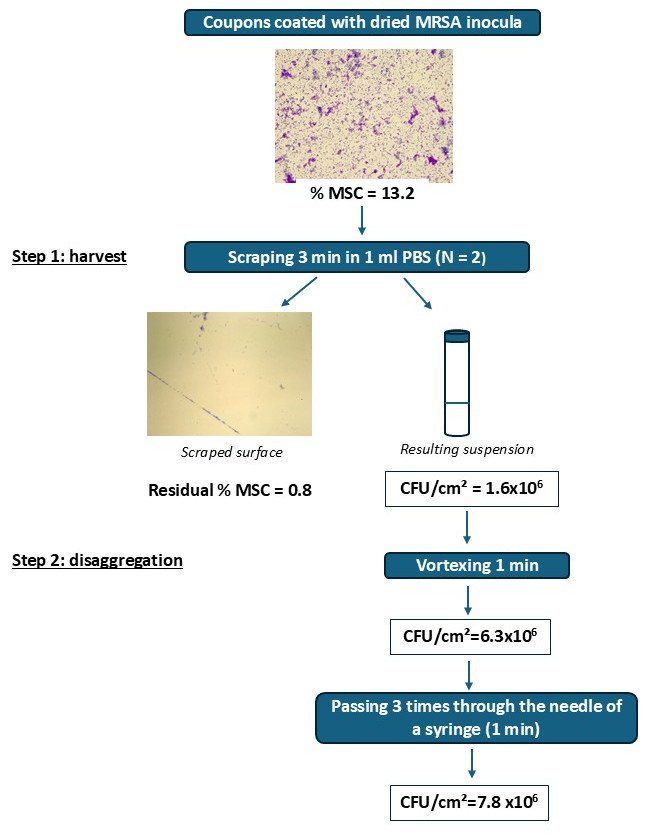
**
